# Editorial: Performance enhancement and injury mitigation in futsal

**DOI:** 10.3389/fspor.2024.1446031

**Published:** 2024-07-02

**Authors:** Bruno Travassos, João Nuno Ribeiro, Tomás T. Freitas, Pedro E. Alcaraz, Konstantinos Spyrou

**Affiliations:** ^1^ Department of Sport Sciences, Universidade da Beira Interior, Covilhã, Portugal; ^2^ Research Center in Sports Sciences, Health Sciences and Human Development (CIDESD), Covilhã, Portugal; ^3^ Portugal Football School, Portuguese Football Federation, Oeiras, Portugal; ^4^ School of Education, Communication and Sports, Polytechnic Institute of Guarda, Guarda, Portugal; ^5^ SPRINT Sport Physical Activity and Health Research & Innovation Center, Rio Maior, Portugal; ^6^ UCAM Research Center for High Performance Sport, UCAM Universidad Católica de Murcia, Murcia, Spain; ^7^ Facultad de Deporte, UCAM Universidad Católica de Murcia, Murcia, Spain; ^8^ Strength and Conditioning Society, Murcia, Spain; ^9^ NAR Nucleus of High Performance in Sport, São Paulo, Brazil

**Keywords:** team sports, performance, training & development, injuries, monitoring

**Editorial on the Research Topic** Performance enhancement and injury mitigation in futsal

Futsal is a team-sport officially authorized by FIFA and is becoming increasingly popular all over the world. It is characterized as a high-intensity intermittent sport that imposes high physical, technical, tactical, and psychological demands on players. The game is played five-a-side, in a 40 × 20 m court, with a 3 × 2 m goalpost and an unlimited number of substitutions. Despite of presenting a lot of similarities with soccer, in which many skills and tactical concepts overlap allowing the transfer of knowledge from one sport to another (1), there are also some particularities in futsal that require the creation of a specific body of knowledge. For example, futsal has its own specific characteristics and rules (e.g., available space, unlimited number of substitutions and stopped clock) the systems of play adopted by coaches or even the number of players involved, and the specific positional roles defined for goalkeepers, defenders, wingers and pivots (2). The aim of this research topic was to build on the existing scientific literature about futsal and to explore new avenues that will help to really understand the specificities of such sport and contribute to the practice of futsal coaching.

From the five manuscripts accepted, one of the studies is related with the influence of the rule of stopped clock in the procedures of monitoring and analysis of performance. The authors investigated the physical and individual technical-tactical performance of elite futsal players using absolute and effective playing time (Spyrou et al. 2023). By comparing the players that played different amounts of time, when the absolute time was considered in comparison with the effective playing time, a bias in the analysis was observed. Thus, it is recommended in futsal, that before visualization, data should be normalized to the effective time of play of players to not biases their analysis and consequently decision-making (Spyrou et al. 2023).

Regarding the issues of monitoring, one study investigated the countermovement jump (CMJ) as a strategy to evaluate futsal players according to positions and levels of competition (Spyrou et al. 2024). The study concluded that the use of CMJ to evaluate and monitor playerś performance of futsal players should not be limited only to jump height but also consider selected eccentric variables that characterize the initial (downward) phase of the jump. Such variables allow a further understanding of the neuromuscular characteristic of players and to detect changes in jump strategy or deviations in technique. In line with the specific demands of the game, the eccentric variables (center of mass displacement, peak force absolute and relative and peak velocity) discriminate professional from semi-professional players. Thus, it is recommended to include these metrics when monitoring and evaluating futsal players’ performance or physical development programs. In opposition to what was expected by the authors, there was no differences in the results of the CMJ analysis between playing positions (Spyrou et al. 2024). This means that despite the different roles of players from different positions, the neuromuscular characteristic of players remains similar. This result reinforces previous research that revealed players in different positions differ in the frequency and type of actions performed, rather than in their individual capacities *per se* (3).

Continuing in the processes of monitoring and to better understand the mechanisms of injuries in futsal, a study was also developed to understand the typology and incidence of injury in players of different competitive levels during the preseason (Marques et al. 2024) Interestingly, the results showed that the elite group had the lowest rate of injury (4.8 injuries per 1,000 h of exposure) compared to the sub-elite (11.8 injuries per 1,000 h of exposure) and amateur (13.9 injuries per 1,000 h of exposure) groups. However, the elite players displayed the highest percentage of injury occurrence (38.5%). In general, the lower limb was the most affected part of the body (30.8%), and the ligament (23.1%) and the muscle (15.4%) injuries were the most prevalent. In applied settings, based on the differentiation of the incidence and type of injuries according to the competitive level, coaches and physiotherapists may prepare more adequate and tailor made strategies for injury prevention according to the available resources and time of exposure.

Considering training strategies, an innovative study was developed to understand the impact of the application of the warm-up combined with photobiomodulation (PBMT) therapy in the performance of futsal players (Santos et al. 2024). The PBMT therapy is a low-level laser therapy and is considered a non-pharmacological ergogenic aid for enhancing physical performance and recovery. Results revealed that the combination of the warm-up and PBMT therapy significantly improved maximal intermittent running performance compared to conditions involving warm-up and placebo, as well as traditional PBMT without pre-warming up. Thus, the former can be considered as an effective strategy for players activation. However, further research should be developed with high level players and with a larger sample size to really understand the impact of such method to improve players' readiness for performance (Santos et al. 2024).

Finally, in terms of training strategies, looking now to the manipulation of training tasks, a study was conducted to analyse the effects of the manipulation of space and number of players on the external and internal load of youth futsal athletes (Gomes et al. 2024). By considering six training exercises in which the space and the number of players were modified the authors manipulated the relative area of play per player in relation to the match. The results revealed that the use of large relative areas (30 × 20 and 40 × 20 m) and low number of players (2- and 3-a-side), increased the relative area per player and, consequently, the external and internal load on futsal players compared to match demands. Conversely, the use of tasks with a high number of players (3- and 4-a-side or more) in medium to small spaces tended to emphasize the development of collective behaviours but with low physical and physiological effects.

Although there were few studies on this research topic, each one opened new routes for the development of more research and, particularly, for improving the practices of coaches and physiotherapists. In conclusion, this research topic has made simple but effective contributions to the development of knowledge in futsal. For the next years, and considering the title of this research topic, we expect that research could be more focused on the understanding of the processes that support players development, integrating the different aspects that concurrently contribute for “*Performance Enhancement and Injury Mitigation in Futsal*”.
